# Evolution of nonstop, no-go and nonsense-mediated mRNA decay and their termination factor-derived components

**DOI:** 10.1186/1471-2148-8-290

**Published:** 2008-10-23

**Authors:** Gemma C Atkinson, Sandra L Baldauf, Vasili Hauryliuk

**Affiliations:** 1 Department of Biology, University of York, Heslington, York, YO10 5DD, United Kingdom,; 2 Department of Evolution, Genomics and Systematics, Uppsala University, Norbyvägen 18C, 752 36 Uppsala, Sweden; 3 Department of Cell and Molecular Biology, Molecular Biology Program, Box 596, Uppsala University, 751 24 Uppsala Sweden; 4 University of Tartu, Institute of Technology, Nooruse St 1, room 425, 50411 Tartu, Estonia

## Abstract

**Background:**

Members of the eukaryote/archaea specific eRF1 and eRF3 protein families have central roles in translation termination. They are also central to various mRNA surveillance mechanisms, together with the eRF1 paralogue Dom34p and the eRF3 paralogues Hbs1p and Ski7p. We have examined the evolution of eRF1 and eRF3 families using sequence similarity searching, multiple sequence alignment and phylogenetic analysis.

**Results:**

Extensive BLAST searches confirm that Hbs1p and eRF3 are limited to eukaryotes, while Dom34p and eRF1 (a/eRF1) are universal in eukaryotes and archaea. Ski7p appears to be restricted to a subset of *Saccharomyces *species. Alignments show that Dom34p does not possess the characteristic class-1 RF minidomains GGQ, NIKS and YXCXXXF, in line with recent crystallographic analysis of Dom34p. Phylogenetic trees of the protein families allow us to reconstruct the evolution of mRNA surveillance mechanisms mediated by these proteins in eukaryotes and archaea.

**Conclusion:**

We propose that the last common ancestor of eukaryotes and archaea possessed Dom34p-mediated no-go decay (NGD). This ancestral Dom34p may or may not have required a trGTPase, mostly like a/eEF1A, for its delivery to the ribosome. At an early stage in eukaryotic evolution, eEF1A was duplicated, giving rise to eRF3, which was recruited for translation termination, interacting with eRF1. eRF3 evolved nonsense-mediated decay (NMD) activity either before or after it was again duplicated, giving rise to Hbs1p, which we propose was recruited to assist eDom34p in eukaryotic NGD. Finally, a third duplication within ascomycete yeast gave rise to Ski7p, which may have become specialised for a subset of existing Hbs1p functions in non-stop decay (NSD). We suggest Ski7p-mediated NSD may be a specialised mechanism for counteracting the effects of increased stop codon read-through caused by prion-domain [PSI+] mediated eRF3 precipitation.

## Background

Members of eRF1 and eRF3 protein families are involved in two major cellular processes in both eukaryotes and archaea. Firstly, these proteins are involved in translation termination [1,2]. Secondly, both eRF1 and eRF3 are key players in mRNA quality control surveillance mechanisms, as are their paralogues Dom34p in the case of eRF1, and Hbs1p and Ski7p in the case of eRF3 [3-6]. Involvement of these proteins in two different cellular systems and differences in substrate specificity among family members make them interesting candidates for *in silico *comparative analyses. Such analyses can provide a direct link between protein sequence and structure as well as insight into functional aspects of translation termination and mRNA decay.

During translation termination, nascent peptide is released from the ribosome by hydrolytic attack of the water molecule, leaving the P-site tRNA in a deacylated state. This is accomplished by the combined action of two distinct functional classes of proteins, the class-1 and class-2 release factors (RFs). Class-1 RFs (eRF1, aRF1, RF1 and RF2) recognise stop codons in the ribosomal A-site and trigger hydrolysis of the peptidyl-tRNA in the peptidyl transferase center (for a review see [7,8]). Class-2 RFs (aRF3 and RF3) are GTPases that assist class-1 RFs in this process.

Eukaryotic and archaeal class-1 RFs (aRF1 and eRF1, respectively) are homologues of each other but not of bacterial class-1 RFs (RF1 and RF2). This is clear from the lack of structural similarity between them [9] as well as functional differences [1,5,10-14]. Meanwhile, Class-2 RFs are found in both eukaryotes and bacteria (but so far not Archaea [15,16]). However, although the latter proteins are members of the translational GTPase (trGTPase) superfamily [14,17,18], they have very different origins within it; the eukaryote protein (eRF3) arises from the a/eEF1A side of the superfamily, hereafter referred to as the EF1 family [16] while the bacterial protein (RF3) arises from the distantly related EF2 side [19]. Consistent with its EF1 origin, eRF3 binds and transports eRF1, a structural mimic of tRNA [20], to the ribosomal A-site, similar to the role of eEF1A in binding and delivering aminoacyl-tRNAs to the same site. The class-1 RFs appear to be essential as a/eRF1 is universal among eukaryotes and archaea. For the class-2 RFs, eRF3 was reported to be an essential protein in eukaryotes [21], although later studies showed that over-expression of eRF1 can restore translation termination activity in an eRF3 temperature sensitive mutant [5]. RF3, on the other hand, is a non-essential protein in bacteria with a patchy phylogenetic distribution [22].

In addition to their role in translation termination, eukaryotic RFs participate in an RNA surveillance pathway called Nonsense Mediated Decay (NMD) [5,23,24]. NMD occurs when a premature stop codon is encountered during translation (for a review see [25]). During NMD, eRF1 and eRF3 are recruited to the ribosome and act as a platform for the assembly of the NMD multi-protein complex on the mRNA. The NMD complex eventually targets the corrupted message for rapid degradation by Dcp1–Dcp2, Xrn1 and the exosome. At the core of the NMD complex are the Upf proteins, which have conserved roles in animals, plants and yeast [26,27]. Upf1 in particular is known to interact with eRF3 in animals and yeast, and its presence in plants suggests eRF3/Upf1p involvement in NMD may have arisen very early in eukaryotic evolution [28].

Alongside NMD, two additional eukaryotic mRNA quality control mechanisms have recently been discovered that involve trGTPases. No-go Decay (NGD) also acts to release ribosomes that are stalled on the mRNA [6]. The onset of NGD in yeast involves the proteins Hbs1p, a eukaryote-specific paralogue of eEF1A and eRF3 in the EF1 family [15,16] and Dom34p (synonym Pelota), a paralogue of eRF1 [29,30]. Acting as a GTP Stabilising Factor (GSF) [31], Hbs1p forms a ternary complex with GTP (Dom34p·Hbs1p·GTP), similar to eRF3 and eRF1 [32,33]. The C-terminus of Hbs1p, which is homologous with eRF3, is sufficient for the Hbs1p·Dom34p interaction, suggesting a similar architecture of complex formation in the two protein pairs [34]. mRNA cleavage is central to NGD, and the RNase responsible is proposed to reside within Dom34p, which has endonuclease activity in the N termini in both the yeast *Saccharomyces cerevisiae *and the archaeaon *Thermoplasma acidophilum *[32,35]. Thus, although more detailed biochemical analysis is required, current data suggests aDom34p carries out its role as an RNase without Hbs1p, just as aRF1 can fulfill its role in termination without a functional eRF3 in archaea and in the eukaryotic *in vitro *translational system [36].

Finally, non-stop decay (NSD), specifically involving another eRF3/Hbs1p homologue Ski7p is so far known only in *S. cerevisiae *[3,4]. This mechanism rescues translating ribosomes that have read through the stop codon instead of terminating. In the proposed model, these ribosomes translate the poly-A tail of the mRNA, adding a poly-lysine tail to the newly synthesised protein. The ribosome then stalls and Ski7p recruits the halted complex for degradation. From complementation experiments, it seems likely that Ski7p functions can be performed by Hbs1p [37], thus it should be specified that when we discuss NSD in the following, we refer specifically to the *Ski7p-mediated *NSD pathway unless stated otherwise.

We have examined the evolution of both class-1 and class-2 RF protein families across eukaryotes and archaea in order to reconstruct evolution of their involvement in mRNA quality control mechanisms. While related or similar mechanisms certainly occur that do not necessarily involve these factors, this study is limited to those involving factors derived from eukaryotic and archaeal class-1 and class-2 release factor families. We predict an absence of peptide release during NGD based on the absence of a characteristic GGQ motif across the entire taxonomic distribution of Dom34p. Involvement of the a/eRF1 NIKS and YXCXXXF motifs in stop-codon recognition is indirectly strengthened by their absence in Dom34p in all examined organisms. Finally, Ski7p-mediated NSD appears to be restricted to a subset of species in the genus *Saccharomyces*.

## Results

In order to investigate the evolution of RNA surveillance mechanisms, we examined the distribution of eRF1/Dom34p and eRF3/Hbs1p family members by extensive database searching. This showed that e/aRF1 and Dom34p are universal among examined eukaryotes and archaea, and confirmed that eRF3 and Hbs1p are so far missing entirely from archaea. Within eukaryotes, eRF3 appears to be universal, while Hbs1p is only missing from several apicomplexan genera. To investigate the molecular evolution of these proteins in more detail, taxonomically broad alignments were created including representatives of all paralogues. Separate alignments were constructed for the two protein families that play central roles in mRNA surveillance and translation termination, eRF1/Dom34p and eRF3/Hbs1p/Ski7p. Consensus sequences based on these alignments were then used to identify regions of sequence conservation for comparison with known structural and functional elements and alignments were used to construct phylogenetic trees.

### eRF1/Dom34p family alignment

**Figure 1:**
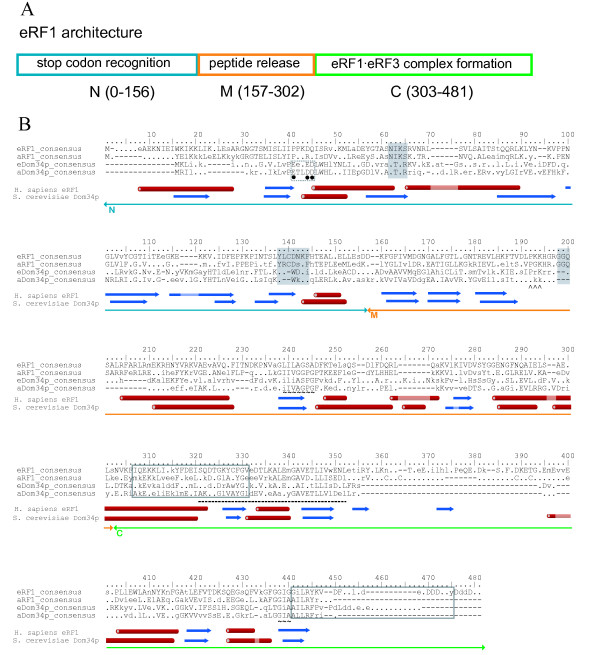
**Architecture and consensus alignments of the eRF1/Dom34p family**. A) A schematic representation of the domain architecture of eRF1 is shown along with B) an alignment of separate consensus sequences for paralogues of eRF1/Dom34p for eukaryotes and archaea. Fifty percent conservation consensus sequences were calculated using the Python program ConsensusFinder (G. Atkinson). Uppercase letters indicate amino acids conserved in > 50% of all examined sequences, and lowercase letters indicate a common amino acid substitution group conserved in > 50% of the sequences. A '.' denotes a position that is universally present but not conserved in sequence, and gaps are denoted by "-". Domains are indicated above the alignment with lines terminating in arrows. Other family-specific symbols are as follows: dashed grey box with filled circles below – location of Dom34p residues implicated in endonuclease activity [32,35], filled grey box – eRF1 structural minidomains, open grey box – human eRF3 binding sites [11], dashed line – RNA binding site [30], "^" characters – putative NLS domain [56], and "~" characters – well conserved patches that fall outside previously reported functional motifs. The a/eRF1-specific insertion at positions 356–393 is shown in detail in additional file 4. Secondary structure is indicated below the alignment for human eRF1 (PDB accession code 1DT9, [91]) and *S. cerevisae *Dom34p (PDB accession code 2VGM, [32]). Blue arrows show β-sheets and red tubes show α-helices. Pale blocks within structural elements indicate positions in the alignment that are not present in the sequence of the protein from which the structure was determined.

eRF1 and Dom34p paralogues contain N (amino-terminal), M (middle) and C (carboxy-terminal) domains (fig. 1A and 1B). In the MAFFT alignment, all three domains are aligned across all eRF1 and Dom34p proteins (fig. 1B). The M and C domains, responsible for eRF3 binding in eRF1 [10,11,38,39], share secondary and tertiary structure conservation, confirming these two domains are homologous across the paralogues [35]. This conservation is also seen at the sequence level, where it is strongest within conserved structural elements (fig. 1B). However, homology is ambiguous in the N domain, particularly the extreme N terminus, where secondary structure is unconserved. The N termini also have different three dimensional folds in eRF1 and Dom34p [35]. Thus, while it is most likely that the N domains of eRF1 and Dom34p have a common evolutionary origin, they have diverged considerably in sequence, structure and therefore probably in function.

#### N domain

*In silico *comparative analyses [40-43], genetic screening [44], domain swapping [45,46], and biochemical studies [20,45,47-49] all support stop codon recognition being carried out in the N domain of eRF1. This activity particularly involves residues in and around the highly conserved NIKS and YXCXXXF motifs (where X represents any amino acid) (fig. 1B). The disruption of either of these motifs has been shown to drastically impair the ability of eRF1 to decode stop signals.

**Figure 2:**
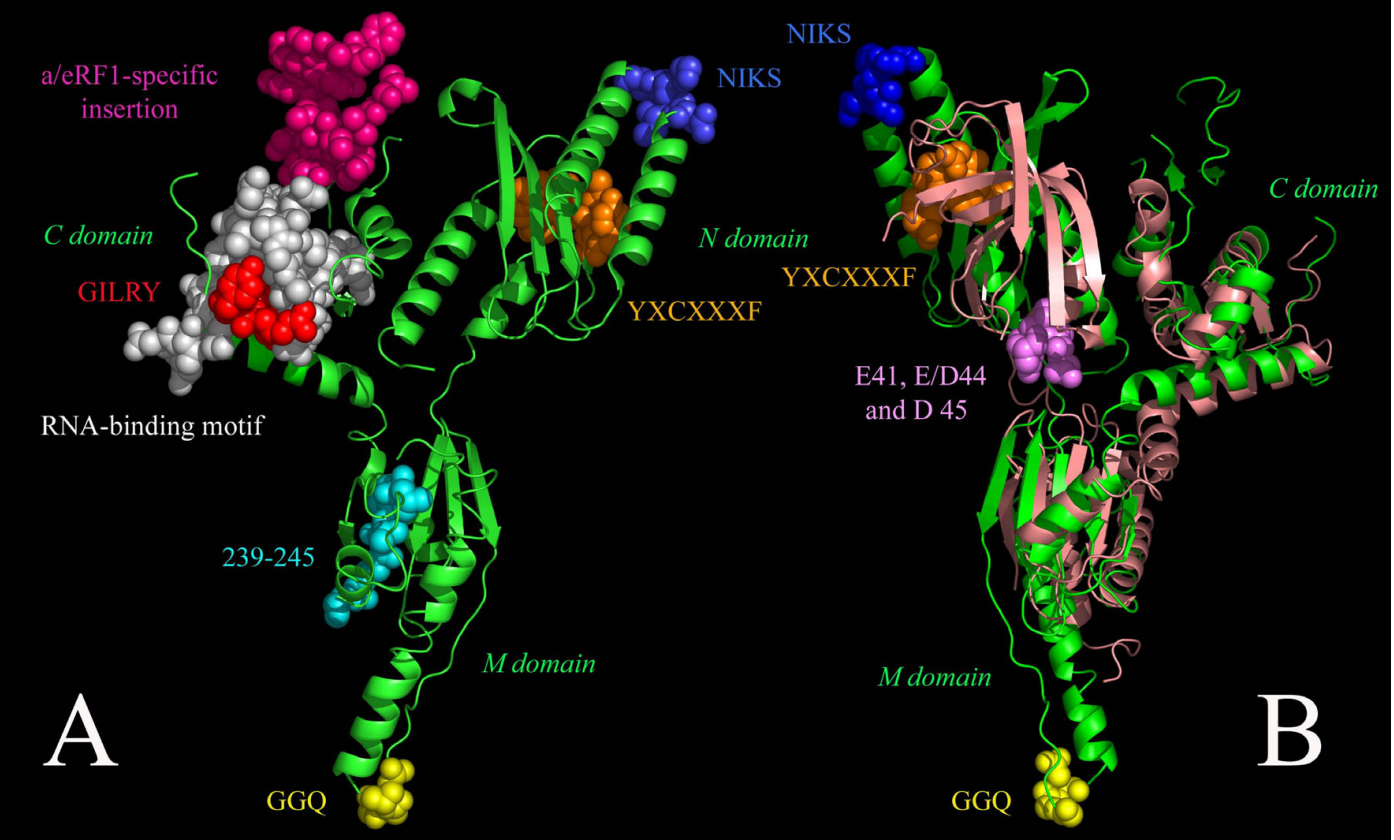
**Annotated structure of human eRF1 alone and superimposed with yeast Dom34p structure**. Panel A: Human eRF1 (PDB accession code 1DT9) structure is shown indicating the location of functional features and patches of residues that are highly conserved across the eRF1 family. Panel B: Superposition of human eRF1 (green) and *S. cerevisae *Dom34p (PDB accession code 2VGM, light pink). eRF1-specific functional motifs are marked with the same color coding as in panel A. Color code and residue coordinates (from figure 2B) are as follows: GGQ motif (yellow, 198–200), NIKS (blue, 62–65) and YXCXXF (orange, 138–144) motifs, GILRY motif (red, 441–445), a/eRF1-specific insertion (magenta, 356–393) RNA-binding motif [30] (white, 320–352), E41, E/D44 and D 45 residues in Dom34p (violet) and M stem conserved region (cyan, 239–245).

Both the NIKS and YXCXXXF motifs are found in the N domain of all e/aRF1 with universal conservation (fig. 1B). However the S of NIKS is not 100% conserved, sometimes present as L, N or D. The only exceptions to the NIK amino acids of the motif are the eRF1s of ciliates as previously reported [40,42]. Two previously unreported ciliate paralogues (eRF1-2s; see below) also have variant NIKS motifs but conserved YXCXXXF and GGQ motifs. *Paramecium tetraurelia *eRF1-2 has 60% identity to its eRF1-1, and these versions have SIQD and SIKN motifs respectively. *Tetrahymena thermophila *eRF1-2 has 31% identity to eRF1-1, and these have SIKN and NIKD motifs respectively. Although they carry the same motif, there is no evidence from the phylogeny that the SIKN versions are orthologous (see below). YXCXXXF is also universal in all sampled a/eRF1 sequences except for a small subgroup of Archaea (*Sulfolobus*, *Caldivirga *and *Hyperthermus*), which carry YXTXXXF. Neither NIKS or YXCXXXF are present in Dom34p (fig 1B), suggesting that the N domain of Dom34p has a substantially different role from that of e/aRF1. Recent crystallographic analysis of *S. cerevisae *[32] and *Thermoplasma acidophilum *[35] Dom34p, shows that the α helical protuberance carrying NIKS in eRF1 is absent in Dom34p and the β-region carrying YXCXXXF has a different architecture in the two proteins (fig. 1B, 2B).

The N domain of Dom34p has been suggested to responsible for the mRNA cleavage that is core to NGD, as this domain in both archaea and yeast has been shown to display endonuclease activity [35]. Positions E41, E/D44 and D45 (fig. 1B, 2B) appear to be important for this function [35]. D45 is particularly well conserved, with all sampled eukaryotic and archaeal Dom34p proteins carrying this residue. D/E41 is 80% conserved across a/eDom34p, and the sole exception to D/E44 is *G. Lamblia*, which has V44, but does have E42 and E43. Thus, this acidic patch is a highly conserved feature of Dom34p in both eukaryotes and archaea, consistent with its involvement in the conserved endonuclease activity in *Thermoplasma acidophilum *and *S. cerevisiae *Dom34p [35]. A conserved role of the N domain in eukaryotes and archaea is also supported by the yeast and archaeal Dom34p N terminal domains being superimposable, although orientation differences mean a conformational change would be required to bring the *S. cerevisiae *protein's N domain into close proximity with mRNA [32].

#### M domain

Induction of peptide release by class-1 RFs in general and e/aRF1 in particular, is completely dependent on a highly conserved GGQ motif in the M domain of the protein [12,50-53] (fig. 1B, fig. 2A). Interestingly, this sequence is also found in the non-homologous bacterial class-1 RF (RF1). RF1 penetrates the ribosomal peptidyl transferase center and positions the GGQ residues adjacent to the CCA-end of the P-site tRNA, thus participating directly in peptide release [54]. Since there is very little sequence similarity otherwise between RF1 and e/aRF1, and their structures are quite different as well [9,55] this appears to be a striking case of convergent evolution. Nonetheless, GGQ is a universally conserved motif in the e/aRF1 M domain (position 198–201, fig. 1B) where it lies at the end of a long protruding arm (fig. 2A).

The GGQ motif is absent from Dom34p (fig. 1B). In fact, in Dom34p, the region corresponding to the e/aRF1 GGQ minidomain is poorly conserved in general including multiple insertions/deletions. This is clearly seen in the superimposition of the eRF1 and Dom34p structures (fig. 2B) [32,35]. The overall organization of the M domain in the two proteins shares the same fold, but the tip of the M domain carrying the GGQ motif in eRF1 is completely absent from Dom34p so that the latter M domain appears truncated by almost 50% relative to eRF1. Although Dom34p from the apicomplexan *Cryptosporidium parvum *contains a GGQ tripeptide in this vicinity, it is difficult to ascertain homology due to the poor sequence conservation and multiple indels in this region (data not shown). However, it is also unlikely that the *C. parvum *Dom34p and eRF1 GGQ motifs are homologous due to the large evolutionary distance between these proteins (see below).

Dom34p and eRF1 also contain a large conserved patch around positions 239–248 (fig. 1B). This region is particularly strongly conserved in aRF1 and eRF1, as indicated by the extent of the consensus sequence. Nonetheless, the exact sequence at this position is not entirely conserved between aRF1, eRF1, aDom34p and eDom34p, such that there is no single universal residue among them. The strongest conservation is residues 244–245 which are PG throughout aRF1, aDom34p and eDom34p. The conservation of this patch suggests it is functionally important, and its location in the M domain suggests that this function may involve positioning the stem of the protein on the ribosome. The 244–245 consensus in eRF1 is the chemically and structurally different motif SA, suggesting an eRF1-specific role of this region in the structure.

eDom34p has been reported to contain a putative nuclear localization signal (NLS), with the sequence PRKRK at coordinates 192–196 (fig. 1B) [29,56,57]. We find a "PrKrr" consensus sequence in eDom34p at this location. This region is at an exposed position in the structure, near the tip of eDom34p and thus would be accessible to nuclear receptors. Similar motifs are also found in both aRF1 (PGKHRk, 192–197) and eRF1 (PKKHGR, 192–197), neither of which are nuclear-localised proteins, strongly questioning the functionality of the putative NLS in Dom34p. This is supported by experimental disruption of this putative NLS [58] which showed that it is dispensable for eDom34p activity *in vivo*.

#### C domain

The most striking feature of the C-terminal domain of eRF1/Dom34p is the GILRY motif (positions 441–445, fig. 1B) implicated in eRF1 eRF3 complex formation [11]. This is actually part of a larger motif with the sequences GFGGIGGILRY in eRF1 and AFGGIAAILRY in aRF1 (fig. 1B). It is surprising that this motif is so conserved in aRF1 since archaea lack eRF3. In fact, the archaeal version of this motif is also well conserved in e/aDom34p, suggesting that its functional role extends beyond eRF3 binding. A second region implicated in eRF1 eRF3 interaction is position 307–331 (fig. 1B, Merkulova et al., 1999). This shows some conservation in e/aDom34p, particularly E309, I/L320, F/Y329 and G330. This stretch overlaps an RNA-binding motif (positions 321–352, fig. 1B, 2A) found in both eRF1 and Dom34p as well as eukaryotic/archaeal ribosomal proteins L30e, L7Ae/S6e and S12 [30]. The conservation of this region across all families, particularly residues A337, A342 and L346, suggests it may have a role in interaction with ribosomal RNA across the paralogues.

Following the GFGGIGGILRY/AFGGIAAILRY motif, eRF1 and eDom34p proteins carry a poorly aligned region that is variable in length and rich in acidic amino acids [16]. This region has been implicated in eRF3 binding by eRF1 [10], although the lack of sequence conservation suggests that composition is more important than sequence in this region. Examination of the 48 available archaeal genomes reveals a similar aRF1 acidic extension in all *Caldivirga *and *Pyrobaculum *species, some *Thermoproteaceae*, and a shorter version in all examined *Ferroplasma *and *Thermoplasma *species (additional file 1).

Region 356–393 (fig. 1B, 2A), contains a large insertion in a/eRF1 relative to a/eDom34p in all examined eukaryotes and Euryarchaea. Where found, this extra sequence is roughly similar in length but there is little sequence conservation, even within eukaryotes. There also appear to have been a number of losses and small indels and the insertion is most notably absent in some Crenarchaea and in *Nanoarchaeum equitans *(additional file 2). This suggests two independent losses may have occurred, one within Crenarchaea and one in the lineage to *N. equitans*, which can not be placed confidently in the phylogeny (see below). The functional significance of this extra sequence is unknown, but it forms a prominent highly exposed protuberance in the structure (fig. 2A).

Across the length of the proteins, a/eRF1 in general displays more sequence conservation than e/aDom34p. This is apparent from the relative extent of their consensus sequences (fig. 1B) as well as in their relative branch lengths in the phylogenetic tree (see below). This suggests that e/aRF1 is under more evolutionary constraint at the sequence level than e/aDom34p.

### Molecular phylogeny of eRF1/Dom34p family

**Figure 3:**
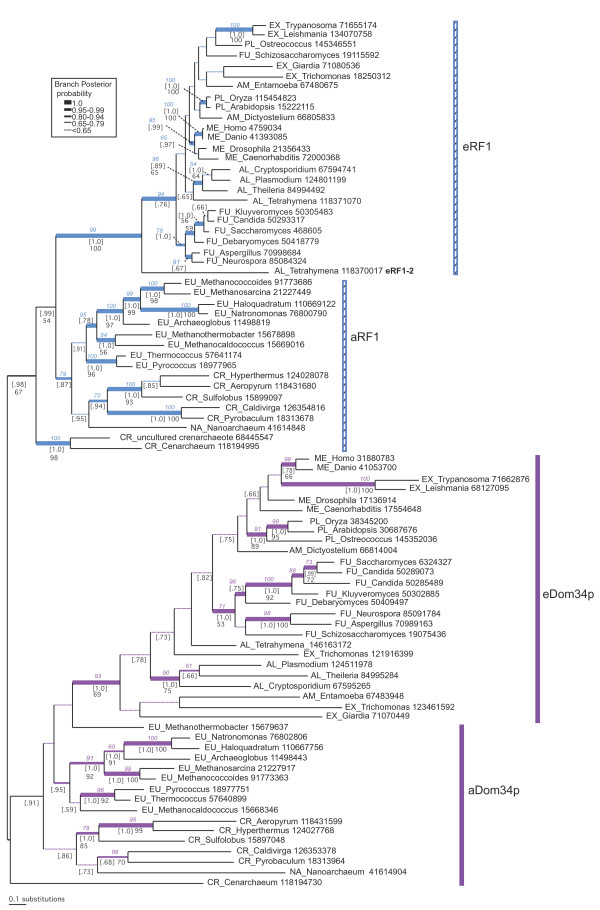
**Phylogeny of the eRF1/Dom34p family**. The tree shown was derived by Bayesian inference based on 178 universally aligned positions of eRF1 and Dom34p amino acid sequences from the M and C domains. The analysis was terminated after 5 million generations, at which point the SDSF was 0.0205 and 500,000 generations were discarded as burn-in. Branch lengths are drawn to scale as indicated by the scale bar at lower left. Branch support values from this analysis are indicated in black, with BIPP in square brackets and MLBP without brackets. Only MLBP values greater than 50% and BIPP values great than 0.65 are displayed. Branch thickness is drawn proportional to BIPP values from separate phylogenetic analyses of full length a/eRF1 (349 positions, blue branches) and a/eDom34p (292 positions, purple branches) as indicated by the key to the right of the figure. Numbers in blue and purple italics correspond to MLBP support from the separate a/eRF1 and a/eDom34p phylogenetic analyses respectively. The tree topologies generated from these separate analyses are shown in additional files 3 and 4. Archaeal and eukaryotic taxon names are preceded by major group designations as follows: ME: metazoa; EX: excavata; FU: fungi; AM: amoebozoa; AV: alveolates; PL: archaeplastida ("plants"); NA: Nanoarchaea; EU: euryarchaeota; CR: crenarchaeaota. Names are followed by NCBI GI numbers.

An unrooted phylogeny of a/eRF1 and a/eDom34p M and C domain sequences from archaea and eukaryotes (fig. 3) shows the universal distribution of these subfamilies in all examined taxa. To gain greater resolution of within subfamily relationships, separate phylogenies of a/eRF1 and a/eDom34p were carried out using the full length alignment, with positions from the N, M and C domains (additional files 3 and 4). The branch support values from these full length analyses are also indicated on figure 3 subtrees. Eukaryotes are reproduced as a strongly supported monophyletic group by both proteins (1.0BIPP, 99% MLBP for full length eRF1 and 1.0BIPP, 93% MLBP for full length eDom34p), but neither aRF1 or aDom34p have support for monophyly of archaea, which are weakly paraphyletic in both subtrees. Although there is limited resolution, particularly in the M+C domain analyses, both proteins seem to be vertically inherited in eukaryotes and archaea as many major groups are reproduced with good support in the independent full length (N+M+C) analyses, particularly with eDom34p (additional files 3 and 4). The improved taxonomic resolution with eDom34p over eRF1 is probably due to the faster evolutionary rate of this protein, with the result that it contains more phylogenetic information (*i.e.*, more variable sites).

Several instances of eRF1 paralogues are found. A second, divergent eRF1 from *Tetrahymena thermophila *is found at a basal position among eukaryotes with strong support (1.0BIPP, 94% MLBP with full length eRF1). This suggests an ancient duplication, but the length of the branch makes it hard to rule out artefactual long branch attraction to the outgroup. The evolution of eRF1 in ciliates is of interest, since some species possess variant genetic codes for decoding stop codons. However, duplication of eRF1 has only previously been described in species of *Euplotes *[59,60]. Phylogenetic analysis of a comprehensive ciliate dataset shows several duplications within ciliates, all of which are inparalogues (additional file 5). The exception to this is the divergent *T. thermophila *eRF1-2, which has an unstable long branch but does not appear to be an orthologue of the extra eRF1 of *Euplotes *or of a previously unidentified second eRF1 in *Paramecium *(additional file 5).

### eRF3/Hbs1p family alignment

**Figure 4:**
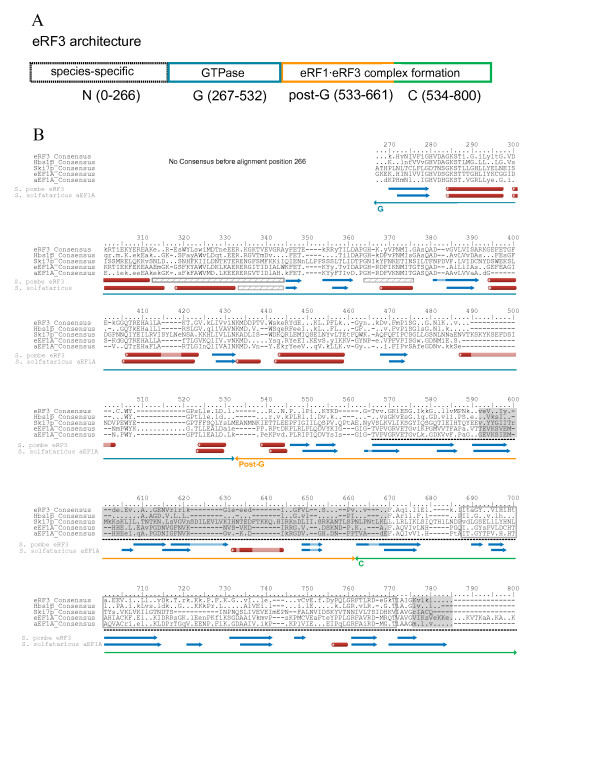
**Architecture and consensus alignments of the eRF3/Hbs1p family**. A) A schematic representation of the domain architecture of eRF3 is shown along with B) an alignment of separate consensus sequences for paralogues of eRF3/Hbs1p families. Sixty percent conservation consensus sequences were calculated using the Python program ConsensusFinder (G. Atkinson). Uppercase letters indicate amino acids conserved in > 60% of all examined sequences, and lowercase letters indicate a common amino acid substitution group conserved in > 60% of the sequences. A '.' denotes a position that is universally present but not conserved in sequence, and gaps are denoted by "-". Domains are indicated above the alignment with lines terminating in arrows. Other family-specific symbols are as follows. Open grey box: *E. octocarinatus *eRF1 binding sites [62], filled grey boxes: human eRF1 binding site [11], dashed line: *S. pombe *eRF1 binding site [61]. Secondary structure is indicated below the alignment for *S. pombe *eRF3 (PDB accession code 1R5B, [65]) and *Sulfolobus solfataricus *aEF1A (PDB accession code 1JNY, [92]). Striped blocked are disordered regions with undetermined structure. Other structural designations are as in Figure 1.

The eRF3/Hbs1p/Ski7p proteins consist of four domains: N (N-terminal), G (GTPase), post-G and C (C-terminal) (fig. 4A, 4B). G, post-G and C-domains are also found in a/eEF1A, while N domains are only found in eRF3, Hbs1p and Ski7p. The N domains are highly variable in sequence and length and may have independent origins in these paralogues. The G domain is highly conserved, typical of all GTPases [19], and the C domain is essential for interactions with eRF1 [11,61,62].

#### N domain

The N-terminal domains of eRF3 and Hbs1p vary greatly in length. In eRF3 its size ranges from being completely absent in *Giardia lamblia *eRF3 [16], to 321 amino acids in *Leishmania majo*r. In Hbs1p, the N-terminal domain of the predicted proteins ranges in size across eukaryotes with extremes of 27 amino acids in *Aspergillus fumigatus *to 367 in *Neurospora crassa*. This variability is consistent with an N-domain role in species-specific regulation of protein activity, as has been shown for eRF3 [63].

The N domains of eRF3 in several species of fungi have repeats rich in Gln, Gly, Asn and Tyr (additional file 6) and has been demonstrated to be prionogenic in some species of yeast [64]. Interestingly, repeats of the same composition are also found in the N terminal extensions of eRF3 in the kinetoplastid protists *Leishmania major *and *Trypanosoma cruzi *(additional file 6). However, given the distant relationship between yeasts and kinetoplastids these repeats are unlikely to be homologous. There is also so far no evidence that the kinetoplastid repeats could be prionogenic.

#### G, Post-G and C domains

Strong conservation is seen throughout domains G, post-G and C (fig. 4B), typical of most members of the EF1 superfamily [15]. Secondary structure is on the whole well conserved between eRF3 and aEF1A (fig. 4B), as has been seen in comparisons of eRF3 with eukaryotic (eEF1A) and bacterial (EF-Tu) orthologues of aEF1A [65]. Conservation is greatest across families in the G (GTPase) domain and drops slightly in the C terminal domain. Characteristic features of all GTPases, such as NKXD and (G/A)XXXXGK(S/T) motifs are clearly visible in the alignment (fig. 4B) (for review see [66,67]). Within this domain, a threonine residue found to be critical for termination activity of eRF3 [68] is universally conserved (position T358, fig. 4B). This residue is conserved across the EF1 superfamily (fig. 4B) reflecting its important structural role in a core β-sheet adjacent to the GTPase switch II (G3) motif that interacts with bound GTP/GDP [69]. The T358 of eRF3 is phosphorylated *in vitro *by a phosphorylation recognition site at alignment position 355–358 in *S. cerevisiae *eRF3 [68] (fig. 4B). However, the functional significance of this is unclear as phosphorylation is not observed *in vivo *[68].

The C terminal domain of eRF3 is responsible for eRF1·eRF3 complex formation. However the exact position for this activity is not known, and evidence from studies in different species seem to differ. In *Schizosaccharomyces pombe *and *S. cerevisiae*, eRF3·eRF1 binding requires the C-terminal one third of eRF3 (positions 566–800, fig. 4B) [61]. In human eRF3, two smaller regions have been implicated (positions 776–785 and 592–669) [11]. In the ciliate *Euplotes octocarinatus*, eRF1 binding has been localized to a region in-between, but not overlapping the human binding sites (positions 688–771) [62]. This could be a result of lineage-specific differences in release factor interactions among eukaryotes. All of these regions have good conservation in eRF3 (fig. 4B), and indeed across the EF1 family. The exception to this is the extreme C-terminal (776- end), the eRF1-interacting decamer in humans [11] which is poorly conserved and variable in length.

The motif GRFTLRD in eRF3 (760–766, fig 4B) is well conserved and postulated to have an important role in eRF1 interactions [65]. Our alignment shows that this patch is also conserved across the EF1 family, especially positions G760, R761 and R765 (fig. 4B). Mutagenesis in *S. pombe *eRF3 identified F762 and R765 as necessary for eRF1 binding [65]. As the residues important for eRF1 binding are also conserved in paralogues of eRF3, they may be important for a more universal EF1 structure or function rather than specific for eRF3 interactions with eRF1.

Ski7p is a divergent variant of Hbs1p. It shows a low overall level of conservation including a striking number of sometimes quite large insertions (fig 4B). Alignment within the Post-G and C domains is especially poor. However, there is enough conservation to confirm homology, especially at positions 644–650, 736–738 and 772–775 (fig. 4B). Quite a number of otherwise universal EF1 family motifs in the Post-G and C-terminal domains appear to be lost completely from Ski7p, including the "GRFTLRD" motif implicated in eRF1 interactions [65] (760–766, fig 4B). Loss of these motifs suggests that some activity common to the other EF1 family members has been lost from Ski7p, although the functional significance of these motifs is unknown.

### Phylogenetic analysis of eRF3/Hbs1p family

**Figure 5:**
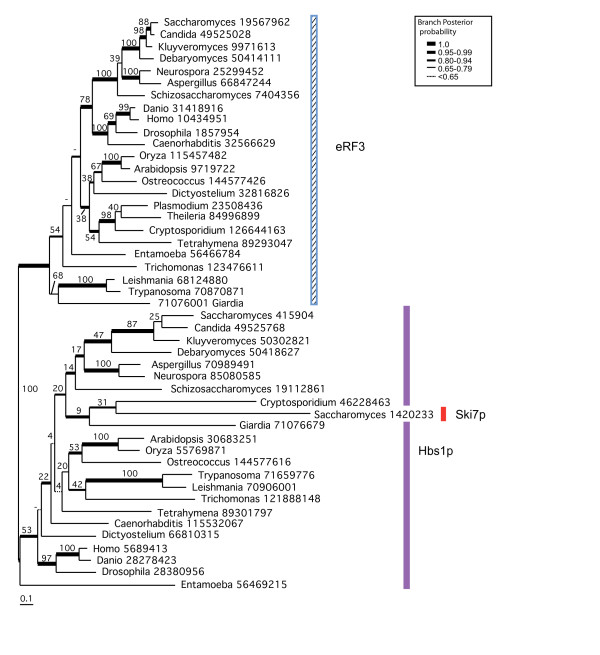
**Phylogeny of the eRF3/Hbs1p family**. The tree shown was derived by Bayesian inference phylogeny based on 395 universally aligned positions of eRF3 and Hbs1p amino acid sequences. The analysis was terminated after 5 million generations, at which point the SDSF was 0.0286, and 500,000 generations were discarded as burn-in. Branch lengths designation, support values and major taxon group designation are as in Figure 4.

Within the eRF3/Hbs1p families eRF3 is universal among eukaryotes, while Hbs1p is nearly universal, missing only from two of the three examined genera of Apicomplexa (*Plasmodium *and *Theileria*; fig. 5). Surprisingly, these apicomplexans still encode the Hbs1p binding partner eDom34p, including the C terminal extension thought to be involved in eRF3 binding to eRF1 [10]. Shorter branch lengths in the tree (fig. 5) and greater conservation across all eukaryotes in the consensus alignment fig. 4B) show that eRF3 generally experiences greater constraint on the primary structure than Hbs1p, as is the case with their binding partners, eRF1 and Dom34p (fig. 1B and 3).

**Figure 6:**
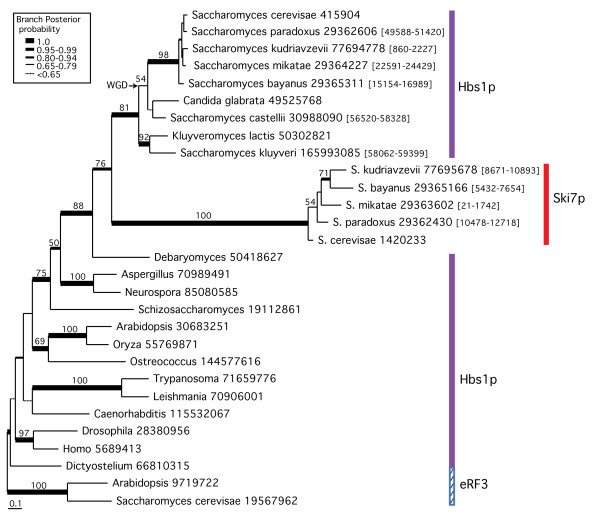
**Phylogeny of the Hbs1p/Ski7p families**. The tree shown was derived by Bayesian inference phylogeny based on 395 universally aligned amino acid positions for Ski7p, Hbs1p, and eRF3 sequences. The tree includes all identified Ski7p sequences. The analysis was terminated after 5 million generations, at which point the SDSF was 0.005, and 500,000 generations were discarded as burn-in. For sequences retrieved as nucleotides and translated into amino acids, the numbers in brackets indicate the start and end coordinates of the genomic DNA that was matched in the TBLASTN search. Branch lengths designation, support values and major taxon group designation are as in Figure 4.

From the alignment (Fig. 4B) and phylogenetic tree (fig. 5), it is clear that Ski7p is a paralogue of Hbs1p, found only in a subset of saccharomycete yeasts. The highly divergent nature of these sequences makes it difficult to place them accurately within a full Hbs1p phylogeny, which includes a number of other long branches (fig. 5). However, without the latter sequences and with the additional *Saccharomyces *Hbs1p and Ski7p identified in genomic TBLASTN searches, the Ski7p sequences form a clade that strongly groups with the rest of the saccharomycete Hbs1p sequences (0.97 BIPP, 88%MLBP, Fig. 6). The Ski7p group appears to arise early within this lineage, possibly after the divergence of *Debaryomyces *(0.97 BIPP, 76%MLBP, fig. 6). Hbs1p and Ski7p have previously been identified as occurring on syntenic blocks in the *S. cerevisiae *genome, suggestive of an origin in the whole genome duplication (WGD) event thought to have occurred in the *Saccharomyces *lineage after the divergence of *Kluyveromyces waltii *[70]. Surprisingly however, the phylogeny in fig. 5 strongly suggests that Ski7p arose from an independent earlier duplication event and was then lost from some lineages (1.0 BIPP, 81%MLBP). The observed synteny may be a result of Hbs1p and Ski7p being adjacent in the genome (following single gene duplication) at the time of the WGD, with subsequent loss of one copy from each of the old and new chromosomal locations. Whenever the precise timing of the origin of Ski7p, this protein clearly arose in ascomycete yeast and in currently available genomes, is limited to a closely related subset of *Saccharomyces *species.

## Discussion

We have analysed datasets of the eRF1/Dom34p and eRF3/Hbs1p/Ski7p protein families in eukaryotes and archaea in order to reconstruct evolution of three different mRNA quality control mechanisms that are known to be governed by these proteins.

We find that Dom34p is universal in eukaryotes and archaea. As the only *biochemically *demonstrated role of Dom34p is in NGD in yeast, and given the sequence, structural and functional similarities between archaeal Dom34p and yeast Dom34p, we propose that NGD is probably an ancient mechanism. We propose that NMD on the other hand is probably restricted to eukaryotes, while NSD specifically mediated by Ski7p is present only in a subset of saccharomycete yeasts. However, NSD mediated by Hbs1p may be more widespread.

Class-1 release factors, such as eRF1, perform two distinct tasks during termination of protein synthesis – recognising the stop codon and then promoting release of the peptide chain. The former requires two motifs, NIKS and YXCXXXF [45,48,49,71], see fig. 2A). We find that the eRF1 homologue Dom34p, which functions in a process devoid of stop codon recognition (NGD), lacks both motifs (see fig. 1B). This is confirmed by the recently published Dom34p structures [32,35] where the whole GGQ and NIKS regions are shown to be absent (fig. 2B).

The absence of the eRF1-specific motifs involved in the stop-codon recognition in Dom34p corroborates well with the available functional information suggesting codon-independence of NGD. This mRNA decay mechanism is onset by ribosome stalls caused by hairpin loops and pseudoknots in addition to rare codons, suggesting that the trigger for NGD is a delay in the onset of elongation rather than the nature of the stall as such [6].

Peptide release by eRF1 is mediated by a GGQ motif at the tip of the M domain (fig. 2A). Release of the nascent peptide greatly destabilises the ribosomal complex, prompting subunit dissociation [72]. The lack of GGQ in Dom34p suggests an absence of peptide release in NGD, with the ribosomal complex remaining stabilised. This may serve to further minimise translation of the NGD-destined mRNA. As Dom34p acts as an endoribonuclease in NGD [35], the stabilised complex may also anchor Dom34p while it degrades the mRNA. It worth mentioning that this stabilisation is transient, since mRNA cleavage in the ribosomal A-site destabilises the ribosomal complex, as seen in experiments with the bacterial toxin RelE. This toxin, similarly to Dom34p, promotes mRNA cleavage in the A-site, destabilising the mRNA and tRNA binding to the ribosome ([72] and VH, unpublished observations).

In yeast, NGD requires the concerted action of Dom34p and Hbs1p. However, while Dom34p is universally present in eukaryotes and archaea, Hbs1p is missing from all examined archaea [15,16] as well as some Apicomplexa. Since the endonuclease activity in NGD appears to reside entirely in Dom34p [35], NGD is most probably present in archaea and Apicomplexa. The absence of both eRF3 and Hbs1p homologues suggests that GTPase participation is dispensable in archaeal NGD as well as in termination. The situation in Apicomplexa is not as easy to explain, but it appears that eDom34p has secondarily acquired the ability to carry out its function without a trGTPase binding partner. As restoration of translation termination in eRF3 temperature sensitive mutants is restored by eRF1 overexpression [5], this suggests that trGTPase involvement is not at the core of NGD or translation termination in eukaryotes. Rather, trGTPase involvement appears to be more peripheral and may be utilised mainly for improving efficiency by delivering the binding partner with the main catalytic function to the ribosome.

In the eRF1 family, three highly conserved patches were found in the M and C domains (239–245, 321–352 and 438–446) (fig. 1B, 2A). All are located on the solvent-oriented face of eRF1 in complex with the ribosome, directly opposite the ribosome-oriented face bearing NIKS and GGQ motifs (see fig. 2A). Positions 321–352 correspond to the RNA binding motif, presumably involved in ribosome binding [30], and positions 438–446 include the GILRY motif, involved in eRF1·eRF3 interaction [11]. Although no specific function has been identified for patch 239–245, its strong conservation across aRF1 and a/eDom34p (fig. 1B) suggests that this region has an important functional role.

Intriguingly, the patches of conserved residues in eRF1 and eDom34p (fig. 1B) that have been implicated in eRF1:eRF3 (and presumably eDom34p:Hbs1p) interaction [10,11,39] are also conserved in aRF1 and aDom34p (fig. 1B). This is despite the fact that archaea lack eRF3 and Hbs1p orthologues. This raises the question of whether aRF1 and aDom34p could be interacting with another eRF3/Hbs1p-like GTPase, the closest candidate for which is aEF1A. However, we find no regions of aEF1A that are obviously shared with eRF3 and Hbs1p to the exclusion of eEF1A (fig. 4) and could therefore indicate retention of a function lost in eEF1A. Additionally, the acidic C-terminal extension that is also crucial for eRF1:eRF3 interaction [10,16] is lacking in all aRF1s except for *Caldivirga *and *Pyrobaculum *(additional file 1). The latter is probably of independent origin and unrelated function. Thus the bulk of the evidence still suggests that a trGTPase binding partner is not required for termination or NGD in archaea.

Although archaea may possess NGD, NMD is almost certainly missing in archaea because they lack eRF3 as well as homologues of other components of the eukaryotic NMD system such as Upf1-3 [28]. eRF3's role in NMD is closely linked to the factor's functional interaction with the mRNA polyA tail. Polyadenylation of mRNA in eukaryotes regulates mRNA stability, efficiency of translation and transport (for a recent review see [73]). In eukaryotic mRNAs, the polyA sequence and polyA-binding protein (PABP) form a complex with the N-domain of eRF3 on the terminating ribosome [24,74,75]. This interaction is implicated in eRF3's functions in regulating polyA deadenylation *via *recruitment of the deadenylation complex [76], stabilisation of the mRNA against the NMD [77] and efficient translation *initiation *[78] and termination [79]. The abovementioned PABP/eRF3 interaction modulates GTP binding to eRF3 [33] which could be one of the signals orchestrating the interplay between the translation termination, initiation, mRNA depolyadenylation and NMD. Thus, since eRF3 is dispensable for translation termination in archaea, the extensive functional connections between eRF3 and the eukaryote-specific mRNA polyadenylation system might be the primary reason for its conservation in this domain of life. In fact, NMD may be a by-product of this interaction. However, it should be mentioned that PABP/eRF3 interactions have only been studied in yeast and animals, where they have been seen to involve the divergent N domain. Thus characterisation of eRF3's role in the polyadenylation system in a wider range of eukaryotes is required to understand further the evolutionary relationship between these processes.

Finally, Ski7p-mediated NSD seems to be unique to a subset of Saccharomycete yeasts (fig. 6). Among sequenced genomes, we find Ski7p only in *S. cerevisae*, *S. kudriavzevii, S. bayanus, S. mikata *and *S. paradoxus*. However, NSD mediated by Hbs1p instead of Ski7p may operate in a wider taxonomic range. Knock-out complementation experiments showing that Hbs1p from *S. kluyveri*, a yeast that does not carry Ski7p, can complement an *S. cerevisiae *Ski7p deletion mutant [37]. This suggests a scenario where Dom34p·Hbs1p complexes act in both NSD and NGD. It is not known at present how widespread Hbs1p-mediated NSD might be in eukaryotes. However, it is tempting to link Ski7p/Hbs1p-mediated NSD with the [PSI+] prionogenic property of eRF3 that is known from several yeast species [64], such as *S. cerevisae *which uses this property to regulate termination potential in the cell. Here, termination efficiency is lowered with the formulation of insoluble eRF3 amyloid fibers in the [PSI+] state or elevated with a transition to the soluble [psi-] state. The PSI+ state can result in up to 16% stop codon read-through [80], which results in longer protein isoforms than are produced in the [psi-] state. However, this also creates a lot of read-through nonsense. Thus, organisms with [PSI+] activity might benefit from an extra mRNA surveillance system that specifically targets read-through messages.

This hypothesis is consistent with the recent finding that Ski7p deletion enhances the observed [PSI+] phenotype [81], which suggests a link between these phenomena. Many yeasts show an eRF3 [PSI+] phenotype caused by N domains rich in Gln, Gly, Asn and Tyr [64]. We find similar N domains in the eRF3 proteins from a range of sampled fungi, including the basidiomycete *Cryptococcus neoformans *(additional file 6). This suggest that [PSI+] activity originated quite early in fungi, before the basidiomycete/ascomycete split. Thus it is possible that the [PSI+] phenotype predates, and perhaps provided the driving force for the specialisation of the Hbs1p duplicate Ski7p in Saccharomycetale NSD. 

## Conclusion

**Figure 7:**
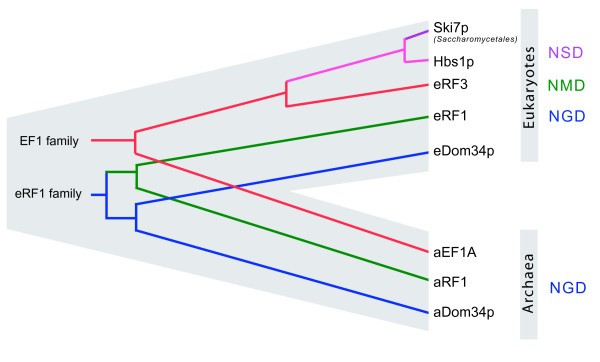
**Evolution of mRNA quality control mechanisms**. A schematic representation of a proposed scenario for the origin and divergence of components of trGTPase-associated mRNA decay mechanisms in archaea and eukaryotes is shown. Abbreviations indicate decay mechanisms known to function in eukaryotes and potentionally functional in archaea as follows: NSD – nonstop decay, NMD – nonsense-mediated decay, and NGD – no-go decay.

Based on these results we hypothesise the following scenario (fig. 7). We propose that the last common ancestor of eukaryotes and archaea possessed Dom34p-mediated NGD. This Dom34p may or may not have required a trGTPase for its delivery to the ribosome (the most likely candidate for which would have to be EF1A). Then, at an early stage in eukaryotic evolution, a duplication of the eEF1A gene occurred. One paralogue became eRF3 and was recruited for the termination stage of protein synthesis, interacting with release factor eRF1. This eRF3-type protein evolved NMD-activity before or after it was again duplicated. This second duplication gave rise to modern eRF3 and Hbs1p, with the latter being recruited for NGD. These two duplication events occurred very early in eukaryotic evolution, well before the last common ancestor of all extant taxa. Finally, a third duplication within ascomycete yeast gave rise to Ski7p, which may have become more specialised for a subset of existing Hbs1p function in NSD. The origin of Hbs1p-mediated NSD is unknown, but we propose that Ski7p NSD may be a specialised mechanism for counteracting the effects of increased stop codon read-through caused by [PSI+] eRF3 precipitation. Biochemical investigation of these mechanisms in a wider range of eukaryotes is required to test these hypotheses. Particularly, verification of NGD function of Dom34p in archaea is required, and further investigation of the taxonomic range of Hbs1p-mediated NSD.

Thus, it seems that the evolution of mRNA decay systems in eukaryotes has been driven by eRF1 and eRF3 gene duplications. Interestingly, these two proteins have experienced a number of additional duplications in eukaryotes. Mammals encode two versions of eRF3, which differ mainly in their N terminal domains [82,83] and plants can encode up to three copies of eRF1 [84]. This could be explained by the existence of a diversity of cellular pathways utilising termination events as check points in mRNA metabolism. This also suggests the existence of additional pathways for regulation of mRNA decay and translational activity in eukaryotes that remain to be discovered.

## Methods

Amino acid sequences of eRF1 and Dom34p were retrieved from the NCBI non redundant (nr) database using BLASTP with *Saccharomyces cerevisiae *query sequences. More divergent members of the aRF1 and aDom34p subfamilies were retrieved using *Sulfolobus solfataricus *query sequences. Preliminary alignments and Neighbour Joining phylogenetic trees were derived using Clustal X [85] to confirm the orthology of various members of the eRF1 and Dom34p families (data not shown). Based on these trees, the dataset was trimmed down to a taxonomically broad subset of archaeal and eukaryotic sequences to form the dataset "eRF1/Dom34p." Additional ciliate eRF1 sequences were added to create dataset "Ciliate eRF1" using sequences identified by a similar BLASTP search limited by taxonomy to Ciliates.

A taxonomically broad set of archaeal and eukaryotic eRF3, Hbs1p, aEF1A, eEF1A and Ski7p sequences was downloaded from a local database (trGTPase database, http://www.GTPbase.org.uk, GCA unpublished). Second copies of eRF3 in mammals, which differ mostly in their N terminal domain sequences [86] were not included. Ski7p and Hbs1p gene sequences from additional *Saccharomyces *species were retrieved from the NCBI Core Nucleotide database using a TBLASTN search limited to "Saccharomycetales" and using *S. cerevisiae *Ski7p and Hbs1p protein sequences as queries. Sequences were translated to amino acids using the Emboss Transeq web application [87].

Sequences were aligned using MAFFT v6.234b with strategy L-INS-I [88]. Consensus sequences were computed with the Python program ConsensusFinder (available upon request from GCA). Large gaps and ambiguously aligned regions in the alignments were excluded from subsequent phylogenetic analyses using Bioedit [89] (additional files 7 and 8). Phylogenetic trees were constructed using Bayesian Inference (B1) with MrBayes 3.1.2 and maximum likelihood (ML) with RAxML-VI-HPC 2.2.3 [90]. MrBayes was run for 5 million generations under a mixed amino acid model with a gamma correction for rate variation among sites. Runs consisted of 2 sets of 1 cold and 3 heated chains, with the output saved ("sampled") every 1000 generations. Consensus trees were calculated after 500,000 generations were discarded as burn-in. Maximum likelihood bootstrapping with RAxML was run with the PROTMIXJTT model with 25 per site rate categories and 100 bootstrap replicates.

Structures were visualized and prepared as figures with PyMOL molecular visualization system http://www.pymol.org using PDB 1DT9 for *H. sapiens *eRF1, and PDB 2VGM for *S. cerevisae *Dom34p.

## Authors' contributions

GCA carried out sequence searching and phylogenetic analysis. VH conceived of the study and carried out structural analysis. GCA and VH participated in the design of the study and drafted the manuscript. SLB helped draft the manuscript and participated in the coordination of the study. All authors read and approved the final manuscript.

## Supplementary Material

Additional file 1 **Comparison of the acidic C-terminal extensions in e/aRF1**. The sequences shown are examples of the extreme C-terminal domains of e/aRF1, including the highly conserved C terminal motif (aligned, gray box) and the acidic C-terminal extensions (unaligned). Acidic amino acids are in bold: aspartic acid (D), glutamic acid, (E), asparagine (N) and glutamine (Q). Taxa group designations as follows: EUK: eukaryote; NA: Nanoarchaea; EUR: Euryarchaeota; CR: Crenarchaeaota. Click here for file

Additional file 2 **An insertion in all eRF1 and some aRF1 sequences**. The sequences shown are examples of sequences encompassing the a/eRF1-specific insertion. Residues shown are 333–431 in figure 2B. Taxa group designations as follows: EUK: eukaryote; NA: Nanoarchaea; EUR: Euryarchaeota; CR: Crenarchaeaota. Click here for file

Additional file 3 **Phylogeny of aRF1 and eRF1 sequences from a full length alignment**. The tree shown was derived by Bayesian inference phylogeny based on 349 universally aligned amino acid positions of eRF1 sequences from domains N, M and C. The analysis was terminated after 5 million generations, at which point the SDSF was 0.005, and 500,000 generations were discarded as burn-in. Branch lengths designation, support values and major taxon group designation are as in Figure 3. BIPP and MLBP values from these analyses are also indicated on Figure 3. Click here for file

Additional file 4 **Phylogeny of aDom34p and eDom34p sequences from a full length alignment**. The tree shown was derived by Bayesian inference phylogeny based on 292 universally aligned amino acid positions of eRF1 sequences from domains N, M and C. The analysis was terminated after 5 million generations, at which point the SDSF was 0.004, and 500,000 generations were discarded as burn-in. Branch lengths designation, support values and major taxon group designation are as in Figure 3. BIPP and MLBP values from these analyses are also indicated on Figure 3. Click here for file

Additional file 5 **Phylogeny of ciliate eRF1 sequences**. The tree shown was derived by Bayesian inference phylogeny based on 349 universally aligned amino acid positions of ciliate eRF1 sequences. The analysis was terminated after 5 million generations, at which point the SDSF was 0.0286, and 500,000 generations were discarded as burn-in. Names in italics are ciliates and duplicate copies are indicated by a 2 preceding the taxon name. Branch lengths designation, support values and major taxon group designation are as in Figure 4. Click here for file

Additional file 6 **Compositional bias in the prionogenic region of the eRF3 N domain**. The sequences shown are those with compositional biases in the extreme N terminus of the eRF3 alignment (not present in the consensus alignment). Amino acids associated with prionogenic activity are in bold: glutamine (Q), asparagine (N), Glycine (G), and Tyrosine (Y). The alignment is in interleaved format. Click here for file

Additional file 7 **eRF1 and Dom34p family datasets**. The sequences represented in the a/eRF1 and a/eDom34p datasets are in aligned Fasta format. The positions used for phylogenetic analyses are indicated with aligned masking sequences, where "X" corresponds to a column used in the phylogenetic analysis and "-" corresponds to an ignored column. Titles beginning with " [R]" and " [D]" are a/eRF1 and a/eDom34p dataset sequences respectively, and numbers in titles are NCBI GI numbers. "Mask: [R] [D]" indicates the positions used in phylogenetic analysis for Figure 3, while "Mask: [R]" and "Mask: [D] show positions used in phylogenetic analysis for additional files 3 and 4 respectively. Titles beginning with " [cil]" indicate sequences belonging to the ciliate eRF1 dataset, with "Mask: [cil]" showing positions used in phylogenetic analysis (additional file 5). Click here for file

Additional file 8 **eRF3 and Hbs1p family datasets**. Sequence format is as additional file 7. Titles beginning with " [3H]" indicate the eRF3/Hbs1p dataset sequences, with the rest of the title being the trGTPbase entry ID, including NCBI GI number. Positions used from the [3H] dataset in phylogenetic analysis for Figure 5 are indicated with the "Mask: [3H]" masking sequence. Titles beginning with " [SH]" indicate the Hbs1p/Ski7p dataset sequences. For sequences retrieved as nucleotides and translated into amino acids, the numbers in brackets at the end of the title indicate the start and end coordinates of the genomic DNA that was matched in the TBLASTN search. Positions from the [SH] dataset used in phylogenetic analysis for Figure 6 are indicated with the "Mask: [SH]" masking sequence. Click here for file
