# Supplementary material for: Evolution of nonstop, no-go and nonsense-mediated mRNA decay and their termination factor-derived components
Source: BMC Evol Biol. 2008 Oct 23;8:290. doi: 10.1186/1471-2148-8-290 (PMC2613156; doi:10.1186/1471-2148-8-290)
Supplement: Additional file 1 — Comparison of the acidic C-terminal extensions in e/aRF1. The sequences shown are examples of the extreme C-terminal domains of e/aRF1, including the highly conserved C terminal motif (aligned, gray box) and the acidic C-terminal extensions (unaligned). Acidic amino acids are in bold: aspartic acid (D), glutamic acid, (E), asparagine (N) and glutamine (Q). Taxa group designations as follows: EUK: eukaryote; NA: Nanoarchaea; EUR: Euryarchaeota; CR: Crenarchaeaota. [file 1471-2148-8-290-S1.pdf]

Eur_Thermococcus_kodakarensis_57641174	GGLGAILRYKI**Q**
Eur_Pyrococcus_furiosus_18977965	GGLGAILRFRI**Q**GV
Eur_Methanococcoides_burtonii_91773686	GGVVAILRF**N**TGI
Eur_Methanosarcina_mazei_21227449	GGIAAILRY**N**TGV
Eur_Haloquadratum_walsbyi_110669122	GGIAGILRYATGI
Eur_Natronomonas_pharaonis_76800790	GGIAGILRYSTGV
Eur_Methanospirillum_hungatei_88604069	GGIAAILRYRTGY
Eur_Archaeoglobus_fulgidus_11498819	GGIAAILRFKP**D**GG**Q**
Eur_Thermoplasma_volcanium_13541419	GGLAAVLRFIRK**DN**V**QN**VM
Eur_Ferroplasma_acidarmanus_126008273	GGLAGILRYVP**ENQ**L**N**I
Na_Nanoarchaeum_equitans_41614848	-GIAAFLYYAS**E**
Cr_Methanothermobacter_thermautotrophicus_15678898	GGIGAILRYRP
Cr_Methanocaldococcus_jannaschii_15669016	KGIAAILRYKIH**Q**
Cr_Cenarchaeum_symbiosum_118194995	GKVAAILRY**N**PGY
Cr_Hyperthermus_butylicus_124028078	KGLVGVLHFRIM
Cr_Aeropyrum_pernix_118431680	GGLAGILRFRISTV
Cr_Sulfolobus_solfataricus_15899097	**N**GIVGKLRYRLY
Cr_Caldivirga_maquilingensis_126354816	GGYVAILATPSWII**EE**W**EN**AS**Q**SLT**Q**
Cr_Pyrobaculum_aerophilum_18313678	GGYVALLSTPVWVL**EQQ**LSIA**E**AA**Q**R
Cr_Pyrobaculum_islandicum_119871817	GGYVALLSTPVWVL**EQQ**I**Q**A**E**AA
Cr_Pyrobaculum_arsenaticum_145591216	GGYVALLSTPVWVL**EQQ**IAA**E**AATTTS
Cr_Pyrobaculum_calidifontis_126458978	GGFVALL**N**APSWLL**EQQQ**TA**Q**A
Euk_Homo_sapiens_4759034	GGIGGILRYRV**D**F**Q**GM**E**Y**Q**GG**DDE**FF**D**L**DD**Y
Euk_Arabidopsis_thaliana_15222115	GGIGGMLRY**Q**L**D**MRTF**DE**LS**D**T**E**VY**ED**S**D**
Euk_Saccharomyces_cerevisiae_468605	GGIGAMLRYKV**N**F**EQ**LV**DE**S**EDE**YY**DEDE**GS
Euk_Dictyostelium_Discoideum_66805833	GGLGGLLRY**Q**V**D**FA**Q**L**ND**F**DN**P**DENE**Y**DD**S**D**S**D**F
Euk_Plasmodium_falciparum_124801199	GGFGGMLRYKI**D**L**N**LY**DED**V**E**S**D**V**E**LF
Euk_Giardia_lamblia_71080536	GGIGGLLRW**Q**V**D**LV**E**AARFM**QE**S**DED**SFM**DD**L**ED**FI
