# Supplementary material for: Evolution of nonstop, no-go and nonsense-mediated mRNA decay and their termination factor-derived components
Source: BMC Evol Biol. 2008 Oct 23;8:290. doi: 10.1186/1471-2148-8-290 (PMC2613156; doi:10.1186/1471-2148-8-290)
Supplement: Additional file 2 — An insertion in all eRF1 and some aRF1 sequences. The sequences shown are examples of sequences encompassing the a/eRF1-specific insertion. Residues shown are 333–431 in figure 2B. Taxa group designations as follows: EUK: eukaryote; NA: Nanoarchaea; EUR: Euryarchaeota; CR: Crenarchaeaota. [file 1471-2148-8-290-S2.pdf]

```
aRF1_Eur_Thermococcus_57641174            EVRKALELG--AVDKLLISEGYDKVRVRAKCNNCGWEELKTM-SEGEFHVYKKQL--THCPKCGSQNITFEKWDVAEELIKMAEESGADVEIISLDTEEGQQ
aRF1_Eur_Pyrococcus_18977965              EVRKALELG--AVDTLLISEGYDKVRVHVKCNNCGWEELKTM-SEEEYEAYKKRI--QTCPKCGSQNLTFEKWEVAEELIKIAEEAGSNVEIISLDTEEGQQ
aRF1_Eur_Methanococcoides_91773686        NVRENLIIG--AVDILLISEDLRAVRETVKCTSCDYEQKTSR-EF---KPGDSSSPTGNCPKCGSYLEITEKVDVVDQLSEICDQMGTRVEFISTDFEEGSQ
aRF1_Eur_Methanosarcina_21227449          QVRANLEIN--SVDVLLLSEDLRAERVTTKCSVCGYENKWTR-RW---KPGEPAPAAGNCPKCGSSLEVTDVTDIVDEFSELADKSNAKVVFVSTDFDEGSQ
aRF1_Eur_Archaeoglobus_11498819           EVRRYIELG--AVDTLLLSEDLRYERVKYRCPKCGKEVEVTV-RE-------GIEKPPFCEEDNVEMEEVERRDIVLELSELAESTGAKVEFLSTESEEGEM
aRF1_Eur_Methanothermobacter_15678898     EVRQHLQMG--AVEVLLLSEDLKYQRGTYECASCGHRMEKTG-RD--------LPDTETCPSCNDQMRLSDRRDMIDDLVEMAEEVGTEVEIISTETEEGMQ
aRF1_Eur_Methanocaldococcus_15669016      EVLEALMMG--AVDTLIVSEELEKYKVKIACNNCDYLEEKTV-NKLELIKLEEELKNAQCPKCGGALSIVEEKDYIEYLSELCEQSGAKLVTVSSETEEGAM
aRF1_Eur_Haloquadratum_110669122          PTRKNLMMG--AVDRLLLSEDLRSDVVVYECPDGHEEYEVID-RR-------HDDPEHTCSDCGSASEKTEREDVIEYLMSIAEQRGTETKFISTDFEKGEQ
aRF1_Eur_Natronomonas_76800790            ATRQNLMMG--SVDRLLISEDLRKDVVTFDC-GSREEYELID-RR-------ADTPDHTCED-GSEGEAVEREDAIDHLMELAEQRGSDVKFISTDFEKGEQ
aRF1_Cr_uncultured_crenarcheote_68445547  DVINALKSG--VADIVLVNDNINRVRVEVVCKRCNAREERIV-SMDKAIQVKQEMISSACSKCSSIDYDVYEQDLIEYMDELAMNTGARVEVISSSTEDGKM
aRF1_Cr_Cenarchaeum_118194995             EVSDLLDRN--VVETLLITDDANLHRVEAKCRRCGNVQDQTV-EGPRVIPVRTGMLGAPCPSCNSTDLEASQRDVVDYFGLVAAKNGAKIEVISGKAEHGMM
aRF1_Cr_Hyperthermus_124028078            MVKTALEMG--ALKYIIVDEERPD---------------------------------------------------ADTLAEEAKKYGTKVYFLSGDLPEAEW
aRF1_Cr_Sulfolobus_15899097               QIKKAMEIG--AIEAIVIHEDSTDKE-------------------------------------------------LEKLAQDAENYGVKVFVVGDEVPEAEW
aRF1_Cr_Aeropyrum_118431680               DVEAALEMG--AVKTLLIHESRED---------------------------------------------------LEEWVEKAKSSGAQVIVVPESLAEAEW
aRF1_Cr_Caldivirga_126354816              NVLNAIEKG--IAKVVLISEDIGE-------------------------------------------------DEIFKLTTMLRGKKIDLVVIPNSVEESLT
aRF1_Cr_Pyrobaculum_18313678              RALKALEMG--IADIIVIAEELGE-------------------------------------------------DAVLEVIMKAEEKGIKVEVVPRGVEESKT
aRF1_Na_Nanoarchaeum_41614848             EVKKYLEQG--LVQKIIVMEDFPK---------------------------------------------------LDEILELAEKHNVDVEIIEGGTTEAEQ
eRF1_Euk_Homo_4759034                     DTLKALEMG--AVEILIVYENLDIMRYVLHCQGTEEEKIL---YLTPEQEKDKSHFTD--KETGQEHELIESMPLLEWFANNYKKFGATLEIVTDKSQEGSQ
eRF1_Euk_Arabidopsis_15222115             DTLKALEMG--AIETLIVWENLDINRYELKNSTTGEMVVK---HFGKDQESDTSNFHD--SETNAELEVQEKMPLLEWFANEYKRFGCTLEFVTNKSQEGSQ
eRF1_Euk_Dictyostelium_6680583            DTLKALDLG--AAHTLIVWESLETIRYLLRLP-TGEEKVI---FLNKDQNKDASVFKD--KESGLDYEIVEEMPIVEWFANNYKNFGASLEFVTNKSQEGSQ
eRF1_Euk_Plasmodium_124801199             DTLKALEIG--AVELLILYEGLDIIRLTTKNPVTNQTKTM---HISPCDEKQESLY----KENNVELEVVEKISLTDWVIGNYKKYGASLDFVTNKSQEGAQ
eRF1_Euk_Saccharomyces_468605             DTLKALDLG--AVEKLIVFENLETIRYTFKDAEDNE--VI---KFAEPEAKDKSFAID--KATGQEMDVVSEEPLIEWLAANYKNFGATLEFITDKSSEGAQ
eRF1_Euk_Giardia_71080536                 DTIRCLEMS--AVEKLIVWDDLPYHRVTLQCVINGETSAPVIKYLLKSQMSNPKYLREVINGEEVQLDIMGDQLLLEWFVDNYKNYGASLEFITNRSAEGTQ
eDom34p_Euk_Saccharomyces_6324327         EVVKAAEYG--AISYLLLTDKVLH------------------------------------------SDNIAQREEYLKLMDSVESNGGKALVLSTLHSLGEE
eDom34p_Euk_Homo_31880783                 QVEKANEAM--AIDTLLISDELFR------------------------------------------HQDVATRSRYVRLVDSVKENAGTVRIFSSLHVSGEQ
eDom34p_Euk_Arabidopsis_30687676          HVEVANERM--AIQTLLITDELFR------------------------------------------NSDVKTRKKYVNLVESVKDSGGDAFIFSAMHVSGEQ
eDom34p_Euk_Dictyostelium_66814004        HVKKANERL--AIETLLVTDELFR------------------------------------------GKDVKTRKKYVDLVQSVKDNKGDVKLFSGLHVTGEQ
eDom34p_Euk_Plasmodium_124511978          EVKYASKIN--AIDSLLITDKTFR------------------------------------------SCDVKTRKEYVQVVQYVKNTGGQVYIFSDNHTSGEQ
eDom34p_Euk_Giardia_71070449              DVLEILKTNPTALSTVLVSTVISQ------------------------------------------GRDLTIRKQIQEYM-AQHSNYIEFIEVKTQTEPGKQ
aDom34p_Eur_Pyrococcus_18977751           EVEEATNYG--AVETLIVLDSLLK---------------------------------------------GELREKIEELMELARNLRASVVVVSSEHEGGDK
aDom34p_Eur_Archaeoglobus_11498443        EVREAHNYR--AIEVLLVADEFLL--------------------------------------------EEREKWDVDGLLREVEESGGKVVIMSTEFEPGKR
aDom34p_Eur_Natronomonas_76802806         EVAEAAEFG--AIETLLILDERLR---------------------------------------EERAGEGDWAVDVNDIIENVEQQGGEVVVFSHEFDPGQQ
aDom34p_Cr_Aeropyrum_118431599            EVLAVARMG--AVDTVLLVDTLLH------------------------------------------SPDDAVREAVDEALRLVESMGGRVIIIPGDSPAGER
aDom34p_Cr_Caldivirga_126353378           EIYEALSMG--AVDSLVMLDKLLI----------------------------------------------EKAEESRRIIDDCIRTRAKLIIVPDGSEAGKL
aDom34p_Cr_Cenarchaeum_118194730          ETRAAAEAG--AIESLVFSDGLIS---------------------------------------------AAGEQQAVDFLNNAQATGAGIFGADSTTDAGLR
aDom34p_Na_Nanoarchaeum_41614904          EAIEDIKIG--NVEEVYVHRDYLK-----------------------------------------RLKERKEEDLLNTILVLPKKYNAKIYIIEGDSDLAKQ
```
