# Supplementary figures and images for: Evolution of nonstop, no-go and nonsense-mediated mRNA decay and their termination factor-derived components
Source: BMC Evol Biol. 2008 Oct 23;8:290. doi: 10.1186/1471-2148-8-290 (PMC2613156; doi:10.1186/1471-2148-8-290)

Branch Posterior  
probability

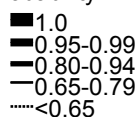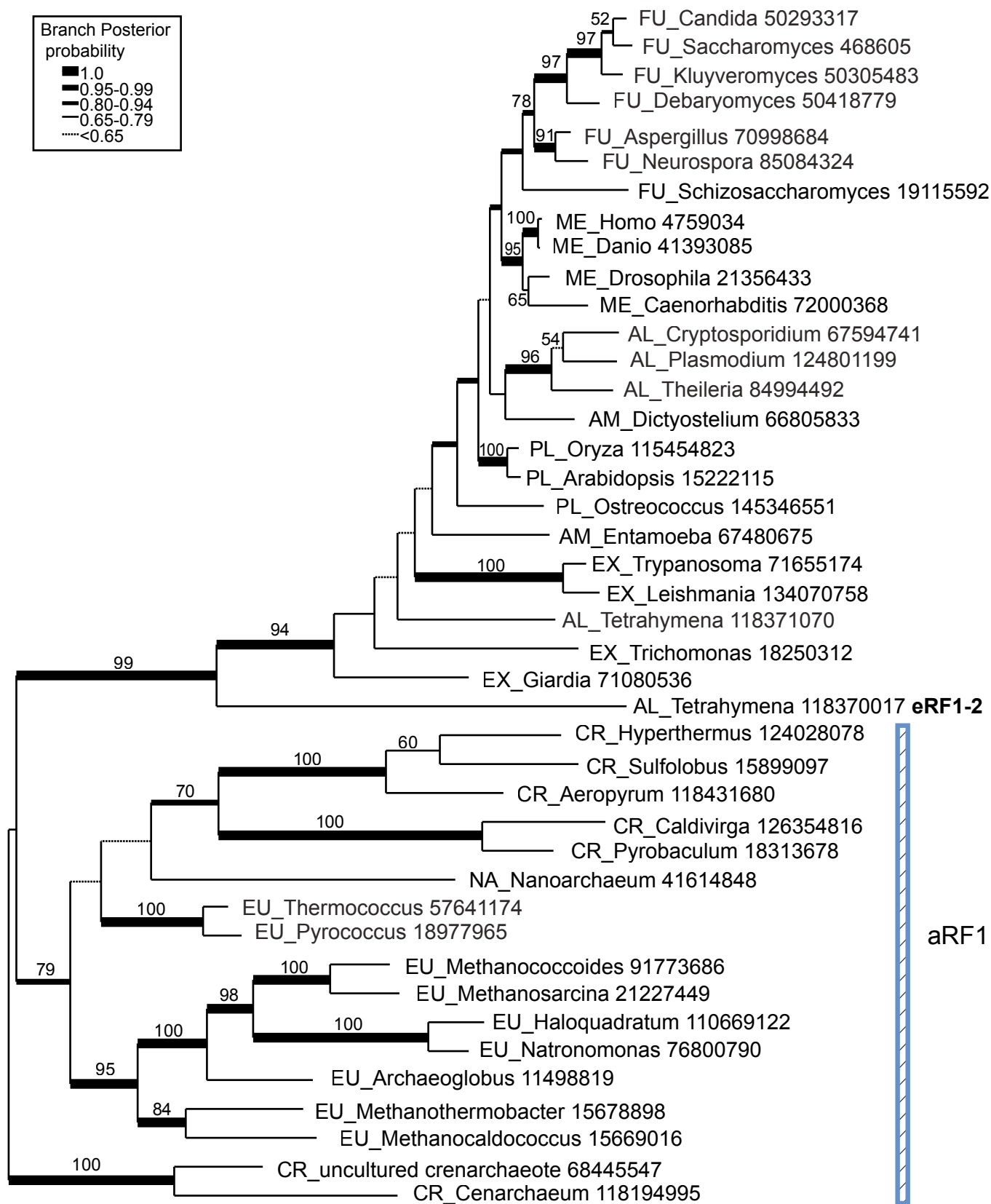

eRF1

aRF1

0.1

Supplement: Additional file 3 — Phylogeny of aRF1 and eRF1 sequences from a full length alignment. The tree shown was derived by Bayesian inference phylogeny based on 349 universally aligned amino acid positions of eRF1 sequences from domains N, M and C. The analysis was terminated after 5 million generations, at which point the SDSF was 0.005, and 500,000 generations were discarded as burn-in. Branch lengths designation, support values and major taxon group designation are as in Figure 3. BIPP and MLBP values from these analyses are also indicated on Figure 3. [file 1471-2148-8-290-S3.pdf]

Branch Posterior  
probability

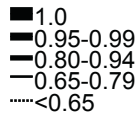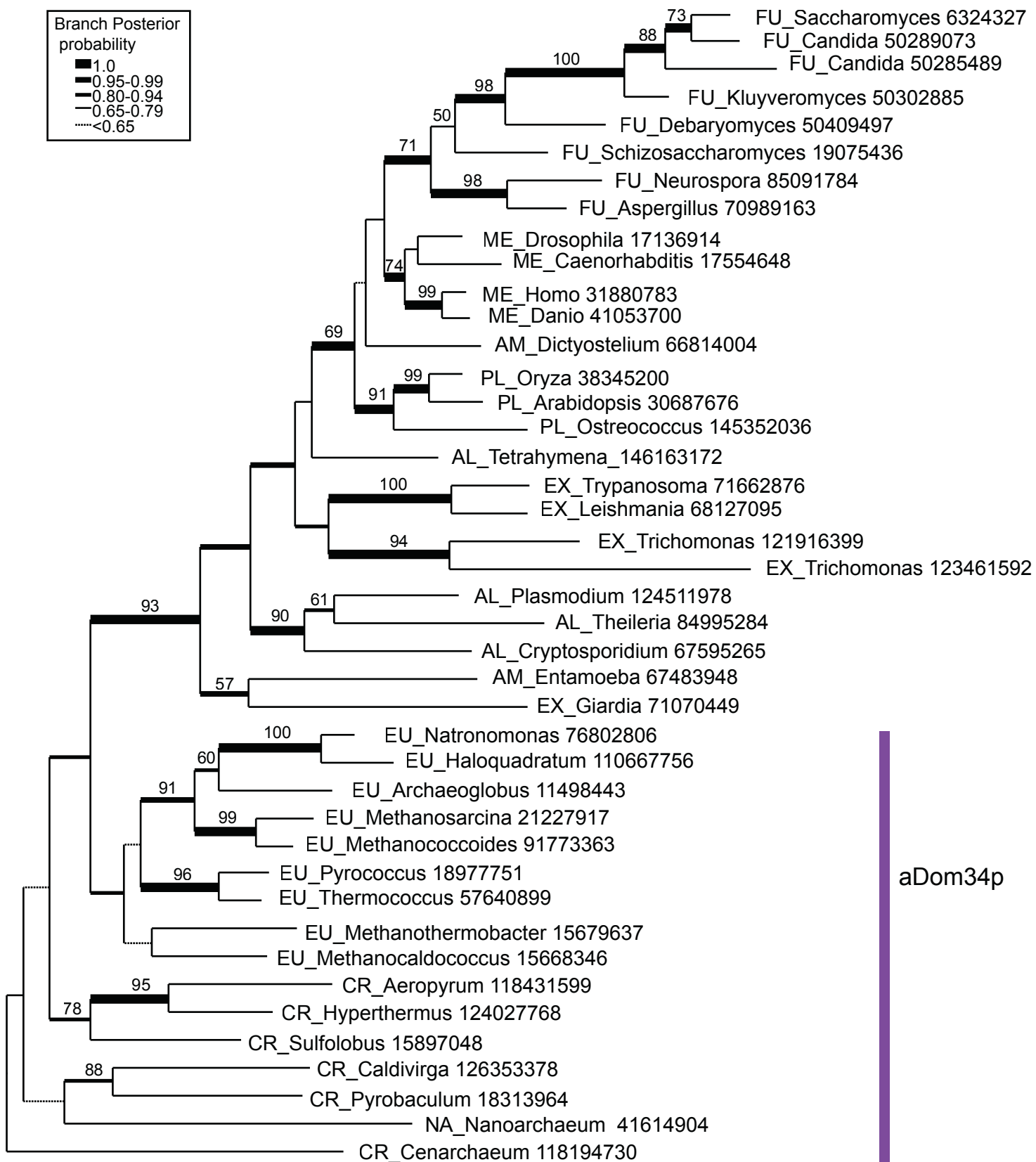

eDom34p

aDom34p

Supplement: Additional file 4 — Phylogeny of aDom34p and eDom34p sequences from a full length alignment. The tree shown was derived by Bayesian inference phylogeny based on 292 universally aligned amino acid positions of eRF1 sequences from domains N, M and C. The analysis was terminated after 5 million generations, at which point the SDSF was 0.004, and 500,000 generations were discarded as burn-in. Branch lengths designation, support values and major taxon group designation are as in Figure 3. BIPP and MLBP values from these analyses are also indicated on Figure 3. [file 1471-2148-8-290-S4.pdf]

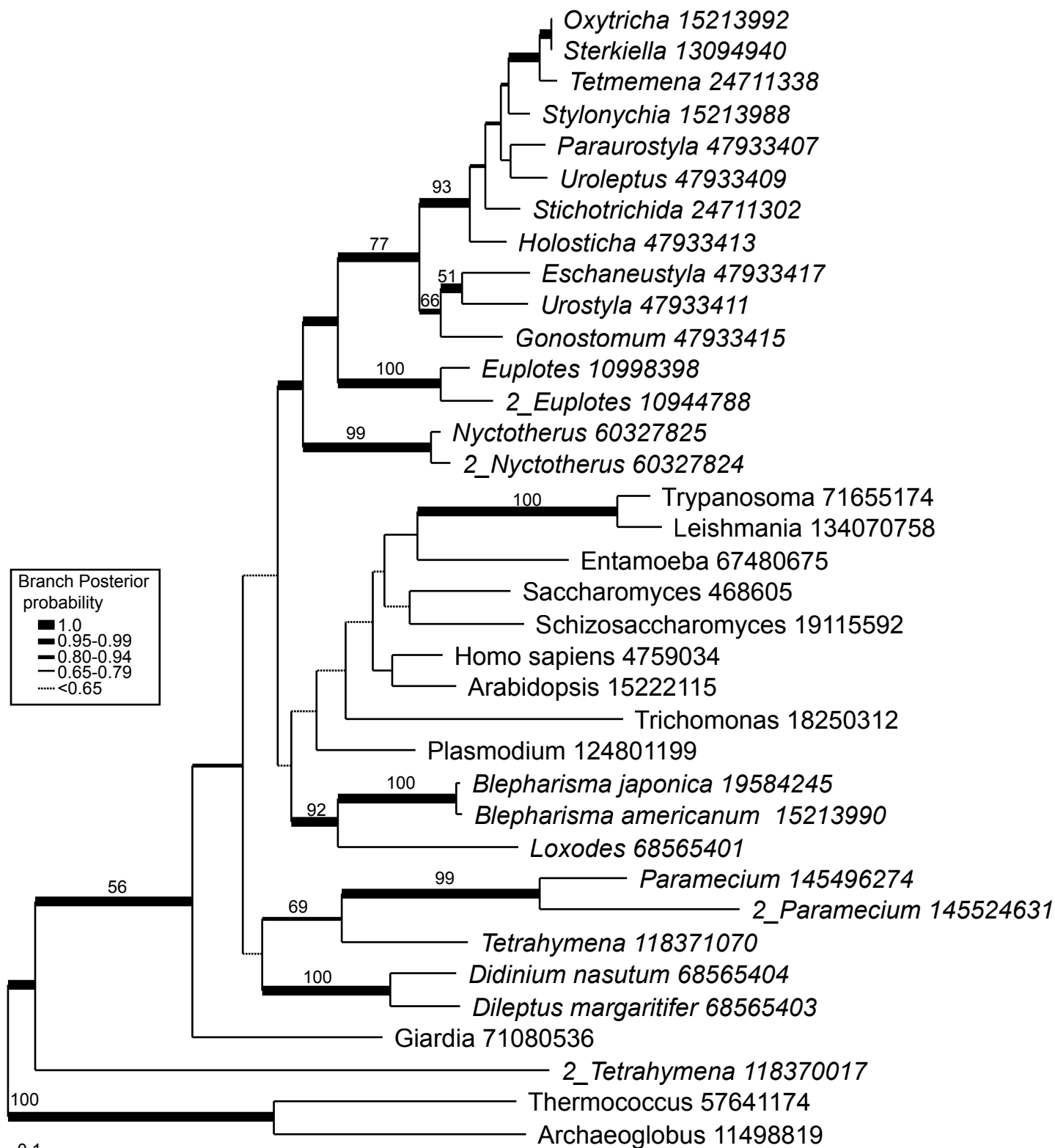

Supplement: Additional file 5 — Phylogeny of ciliate eRF1 sequences. The tree shown was derived by Bayesian inference phylogeny based on 349 universally aligned amino acid positions of ciliate eRF1 sequences. The analysis was terminated after 5 million generations, at which point the SDSF was 0.0286, and 500,000 generations were discarded as burn-in. Names in italics are ciliates and duplicate copies are indicated by a 2 preceding the taxon name. Branch lengths designation, support values and major taxon group designation are as in Figure 4. [file 1471-2148-8-290-S5.pdf]
