# Supplementary material for: Evolution of nonstop, no-go and nonsense-mediated mRNA decay and their termination factor-derived components
Source: BMC Evol Biol. 2008 Oct 23;8:290. doi: 10.1186/1471-2148-8-290 (PMC2613156; doi:10.1186/1471-2148-8-290)
Supplement: Additional file 6 — Compositional bias in the prionogenic region of the eRF3 N domain. The sequences shown are those with compositional biases in the extreme N terminus of the eRF3 alignment (not present in the consensus alignment). Amino acids associated with prionogenic activity are in bold: glutamine (Q), asparagine (N), Glycine (G), and Tyrosine (Y). The alignment is in interleaved format. [file 1471-2148-8-290-S6.pdf]

```
eRF3_Saccharomyces_19567962         MSDSN----QGNNQQNYQQYSQNGNQQQGNNRY--------QGYQAYN--
eRF3_Candida_49525028               MSDPN----QQQQQQQQQQQQQQGNYQQYYQNYGQQNFQPQQGYQQYQQF
eRF3_Kluyveromyces_9971613          MSDQQ--NQDQGQGQGYNQYNQYGQYNQYYNQQGYQGYNGQQGAPQGYQA
eRF3_Debaryomyces_50414111          MSDDQ-QYNQDKLSQDFQNTSIGSGEQQQQSYQQYQQQPQQNNFNANSAP
eRF3_Cryptococcus_58268658          MSGQQ----------------------------PPSFNPGAFEFRPGQAP
eRF3_Neurospora_25299452            MSGNV---QNNWEEAADQDERLARQTQQQMNINAGTFRPGAAAFTPGAPS
eRF3_Aspergillus_66847244           MANQTPDSWEDELSKQTEGVNLNARGQYRPQAQAPSFHPGAASFQPGAPS
eRF3_Schizosaccharomyces_7404356    MASNQ-------------PNNGEQDEQLAKQTSKLSMSAKAPTFTPKAAP
eRF3_Leishmania_68124880            MSWQQPTGNVNPNASSYTPDGGAYINSYNNQEARGYYSPPQQYGGGGYYP
eRF3_Trypanosoma_70870871           MSGWAQPGGFNPNANAYNPQGQQGYYPPQQQQPQGGGYGGQQGRGGYGGY

eRF3_Saccharomyces_19567962         ---AQAQPAGGY--YQNYQ--GYSGYQQGGYQQYNPQGGYQ---QQFNPQ
eRF3_Candida_49525028               ---QNYQPQQGYQQYQNYQQGGYQNYQQGGYQNYQ-QGGYQGGRGGYQGG
eRF3_Kluyveromyces_9971613          YQAYGQQPQGAYQ--------GYNPQQAQGYQPYQGYNAQQQGYNAQQGG
eRF3_Debaryomyces_50414111          TFTPSGQQGGYQGGYQGGYQGGYQNYSQGGYQNYNQGYQNYNQGYQGYQN
eRF3_Cryptococcus_58268658          FAPRQQQPFDPYGQQQGGYPQYGQYGQQQGYPQYGQYGGYP------QQQ
eRF3_Neurospora_25299452            FTPGQFAAPGFTPQYQQQYYGGAQQGYGGGYPQYGQQGYGQ------YNN
eRF3_Aspergillus_66847244           FVPGQTYQQYGGGYPQYGQYGGYPAYDQQQQGFGQYGAYAQ------QPG
eRF3_Schizosaccharomyces_7404356    FIPSFQRPGFVPVNNIAGGYPYAQYTGQGQNSNSPHPTKSYQQYYQKPTG
eRF3_Leishmania_68124880            PQGGLGGNHQGGYANRSNYQGSMYNPQQHRQSVYQDPRNDQGGYYPRGGY
eRF3_Trypanosoma_70870871           SGAYYPPQQFQQPQFGGYSGAYYPPQQQHQQPQFGGYSGAYYPPQQQHQQ

eRF3_Saccharomyces_19567962         GGRGNYKNF----NYNNNLQGYQAGFQP-------------Q--SQGMSL
eRF3_Candida_49525028               RGRGGYKNYNNRNNYNNQNSGYQNYQQQ-------------QQPAQGMTL
eRF3_Kluyveromyces_9971613          HNNNYNKNYNNKNSYNNYNKQGYQGAQGYNAQQPTGYAAPAQSSSQGMTL
eRF3_Debaryomyces_50414111          NNRGGYNNYNNRGGYNNYNNYNQQDQQP--------------VQNQGMSL
eRF3_Cryptococcus_58268658          GYPVPGAPGAGPRAYQPPQARNVQGFQPPSFSSSPAPPPDTKAPAGKPVS
eRF3_Neurospora_25299452            QQQQGYGAVYGQQGYNQGYGQQQQQQQ----QQQYGGYQQNQGYQQRQQQ
eRF3_Aspergillus_66847244           GYNQIYNNQYGGYNQHQQQQYTQPPRQAAPVATQAPSAPAQ---------
eRF3_Schizosaccharomyces_7404356    NTVDEDKSRVPDFS------------------------------------
eRF3_Leishmania_68124880            GGPQQQQGGYSANASYYQGGGYGGQQQQQQQQQCYQQNEAYQQQMQQQLT
eRF3_Trypanosoma_70870871           PQGEYAGHSGGHASQPPVDNIGNTAPQKQQDQQ-----------------

eRF3_Saccharomyces_19567962         NDFQKQQKQ------------
eRF3_Candida_49525028               DSFQQQQEQKS----------
eRF3_Kluyveromyces_9971613          KDFQNQQGS------------
eRF3_Debaryomyces_50414111          ADFQKQQNAQ-----------
eRF3_Cryptococcus_58268658          LSIGGGGAPKAAPSLSIGGGG
eRF3_Neurospora_25299452            NRDAPKPAPQIVKRPEQPAAQ
eRF3_Aspergillus_66847244           ---------------------
eRF3_Schizosaccharomyces_7404356    ---------------------
eRF3_Leishmania_68124880            LQQQQQQQQQQQQKQQK----
eRF3_Trypanosoma_70870871           ---------------------
```
